# Cognitive impairment and autistic-like behaviour in SAPAP4-deficient mice

**DOI:** 10.1038/s41398-018-0327-z

**Published:** 2019-01-16

**Authors:** Claudia Schob, Fabio Morellini, Ora Ohana, Lidia Bakota, Mariya V. Hrynchak, Roland Brandt, Marco D. Brockmann, Nicole Cichon, Henrike Hartung, Ileana L. Hanganu-Opatz, Vanessa Kraus, Sarah Scharf, Irm Herrmans-Borgmeyer, Michaela Schweizer, Dietmar Kuhl, Markus Wöhr, Karl J. Vörckel, Julia Calzada-Wack, Helmut Fuchs, Valérie Gailus-Durner, Martin Hrabě de Angelis, Craig C. Garner, Hans-Jürgen Kreienkamp, Stefan Kindler

**Affiliations:** 1 Institute for Human Genetics, University Medical Centre Hamburg-Eppendorf, 20246 Hamburg, Germany; 2 Behavioral Biology, Centre for Molecular Neurobiology Hamburg (ZMNH), University Medical Centre Hamburg-Eppendorf, Hamburg, Germany; 3 Institute for Molecular and Cellular Cognition, ZMNH, University Medical Center Hamburg-Eppendorf, Hamburg, Germany; 4 Department of Neurobiology, University of Osnabrück, 49076 Osnabrück, Germany; 5 Developmental Neurophysiology, Department of Neuroanatomy, University Medical Centre Hamburg-Eppendorf, Hamburg, Germany; 6 Transgenic Mouse Facility, ZMNH, University Medical Center Hamburg-Eppendorf, Hamburg, Germany; 7 Morphology and Electron Microscopy, ZMNH, University Medical Center Hamburg-Eppendorf, Hamburg, Germany; 8 Behavioral Neuroscience, Experimental and Biological Psychology, Faculty of Psychology, Philipps-University of Marburg, 35032 Marburg, Germany; 9 German Mouse Clinic, Institute of Experimental Genetics, Helmholtz Centre Munich, German Research Centre for Environmental Health, 85764 Neuherberg, Germany; 10 Chair of Experimental Genetics, School of Life Science Weihenstephan, Technische Universität München, 85354 Freising, Germany; 11 German Center for Diabetes Research (DZD), Neuherberg, Germany; 12 German Centre for Neurodegenerative Diseases (DZNE), c/o Charité University Medical Centre, 10117 Berlin, Germany

## Abstract

In humans, genetic variants of *DLGAP1-4* have been linked with neuropsychiatric conditions, including autism spectrum disorder (ASD). While these findings implicate the encoded postsynaptic proteins, SAPAP1-4, in the etiology of neuropsychiatric conditions, underlying neurobiological mechanisms are unknown. To assess the contribution of SAPAP4 to these disorders, we characterized SAPAP4-deficient mice. Our study reveals that the loss of SAPAP4 triggers profound behavioural abnormalities, including cognitive deficits combined with impaired vocal communication and social interaction, phenotypes reminiscent of ASD in humans. These behavioural alterations of SAPAP4-deficient mice are associated with dramatic changes in synapse morphology, function and plasticity, indicating that SAPAP4 is critical for the development of functional neuronal networks and that mutations in the corresponding human gene, *DLGAP4*, may cause deficits in social and cognitive functioning relevant to ASD-like neurodevelopmental disorders.

## Introduction

Throughout life, several dynamic processes control the morphology, number and strength of brain synapses. Alterations in these parameters are mediated through changes in the molecular composition of synapses and via chemical modification of synaptic proteins. Excitatory brain synapses are characterized by the so-called postsynaptic density (PSD), a dense array of more than a thousand different proteins, including transmembrane receptors and adhesion molecules, scaffold and adapter proteins, cytoskeletal components and signalling molecules that together form a dynamic macromolecular complex^1–4^. Tight control of the molecular composition of PSDs is required to establish and maintain proper functional brain circuitry underlying cognition and behaviour. The PSD core contains several modular scaffold proteins of four different families, the Dlg/PSD-95/SAP90 (here collectively referred to as Dlg), SAPAP/GKAP (referred to as SAPAP), Shank/ProSAP (referred to as Shank) and Homer family. Dlg proteins directly associate with ionotropic glutamate receptors (iGluRs) and SAPAPs link these complexes to Shank proteins. The latter build a second molecular layer within PSDs and interact with microfilaments and Homer proteins, which then associate with metabotropic glutamate receptors (mGluRs). This physical link appears to facilitate a functional crosstalk between iGluRs and mGluRs and creates a central platform that organizes the molecular composition of and the signalling within the PSD.

In humans, mutations in genes encoding synaptic proteins have been linked with neurodevelopmental disorders, suggesting that the phenotypic expression of these disorders is, at least in part, attributed to dysfunctions of synapses and neuronal networks^2,5–10^. Affected proteins include presynaptic components, trans-synaptic adhesion/signalling molecules, postsynaptic scaffold modules and regulatory proteins. Within the group of PSD scaffolds, genes encoding Shanks have raised much interest, as it was suggested that mutations in *SHANK1-3* are found in about 1% of all patients affected by ASD^2,10,11^.

Intriguingly, genetic variants of *DLGAP1-4*, encoding SAPAP1-4, have been linked with different neuropsychiatric conditions, including ASD, thus implicating these PSD scaffold proteins in the etiology of these disorders^9,12–19^. After birth, *Dlgap1-4* are broadly expressed throughout the rodent brain with some regional differences^20,21^. While the structural homology between SAPAPs implies some degree of functional redundancy^22^, it is currently unclear whether they perform similar tasks at different synapses or have evolved to assemble functionally distinct synaptic signalling complexes operating in parallel at single synapses. First clues to these issues are emerging from knockout mouse studies. Thus, SAPAP3-deficient mice exhibit a phenotype reminiscent of obsessive-compulsive disorder (OCD) in humans that could be attributed to the specific expression of SAPAP3 in the striatum^23^ and an enhanced synaptic signalling mediated via mGluRs^24,25^. In contrast, the loss of SAPAP2 enhances social dominance and aggressive behaviour^26^, while SAPAP1-deficient mice display reductions in sociability^27^. Also, the loss of SAPAP2 and SAPAP3, respectively, exerts distinct effects on synaptic structure and transmission^23,25,26,28^. Here, we sought to further broaden our understanding of this important family of PSD components. We generated and characterized SAPAP4-deficient mice to specifically examine the relevance of this postsynaptic scaffold protein for the functional integrity of synapses and neuronal circuits underlying social behaviours and cognitive functioning.

## Results

### Generation of SAPAP4-deficient mice

**Fig. 1:**
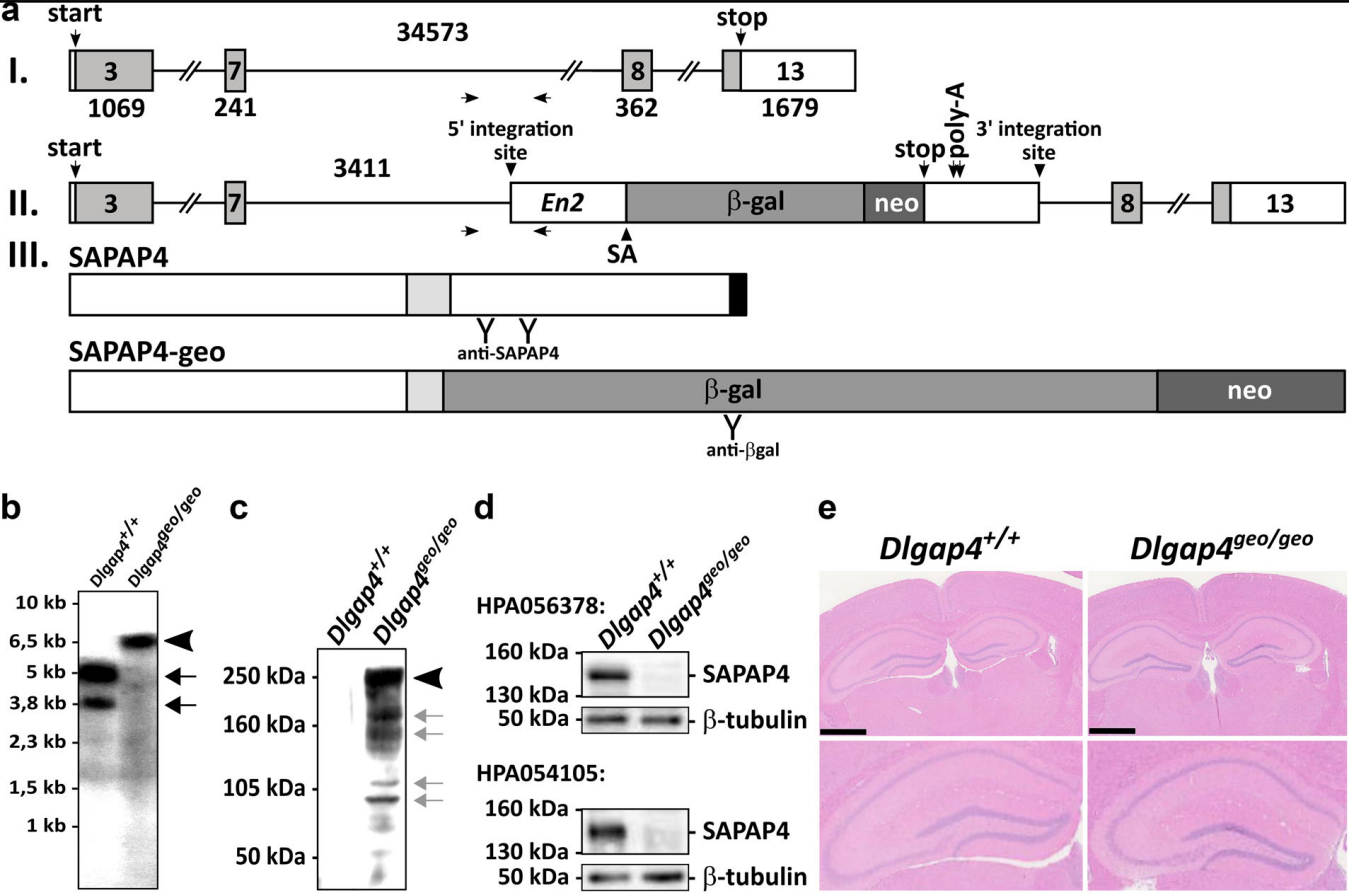
Generation of SAPAP4-deficient mice. **a** Schematic representation of mouse *Dlgap4* gene (**I**) and the corresponding allele disrupted via gene trap technology (**II**). **aI** The 992 amino acid residues spanning protein SAPAP4 (UniProt entry number B1AZP2, schematically shown in (**aIII**)) is encoded by an open reading frame in exons 3–13 (light grey area in numbered boxes). Translation start and stop codons are labelled. Numbers above introns (simple lines) and below exons (boxes) indicate their length in base pairs. Vertical arrows specify the hybridization position of oligonucleotides used for routine genotyping. **aII** In embryonic stem cell clone XH723 (BayGenomics), an exon trap vector is integrated in intron 7 of the *Dlgap4* gene 3411 nucleotides downstream of exon 7. Its 5′ sequence consists of an intron derived from the mouse *En2* gene (white box) harbouring a strong splice acceptor site (SA). This is followed by an open reading frame encoding a fusion protein consisting of β-galactosidase (β-gal, grey box) and neomycin phosphotransferase (neo, dark grey box) as well as two downstream polyadenylation signals (poly-A). **aIII** Schematic representation of mouse SAPAP4 containing binding sites for Dlgs (light grey box) and Shanks (black box), respectively. Epitopes recognized by SAPAP4 and β-gal specific antibodies utilized for Western blotting experiments are marked (Y). The predicted 209 kDa SAPAP4-β-gal-neo fusion protein (SAPAP4-geo) synthesized from the disrupted allele (*Dlgap4*^*geo*^) consists of the N-terminal 549 amino acid residues of SAPAP4 fused to β-gal and neo (geo) (1872 amino acid residues). **b** Northern blot analysis utilizing total RNA obtained from mouse brains. The ^32^P-labelled probe hybridizing to nucleotides #423–1186 (encoding amino acid residues #74-327, NP_775168) of rat SAPAP4 mRNAs (NM_173145) detects wild-type transcripts of about 5.0 kb and 3.8 kb in *Dlgap4*^+/+^ animals (arrows) and the predicted about 6.5 kb SAPAP4-geo fusion mRNA in *Dlgap4*^geo/geo^ mice (arrowhead). **c**, **d** Western blot analysis of PSD fractions obtained from *Dlgap4*^geo/geo^ and *Dlgap4*^+/+^ mouse brains. **c** An anti-β-gal antibody specifically detects the SAPAP4-geo fusion protein present in *Dlgap4*^geo/geo^ but not *Dlgap4*^+/+^ mouse brains (arrowhead). Smaller immuno-reactive proteins may represent breakdown products (grey arrows). **d** Two antibodies recognizing epitopes residing in the C-terminal half of full-length SAPAP4 (amino acid residues 608–691 (Sigma HPA056378) and 692–772 (Sigma HPA054105) of mouse SAPAP4 (NM_146128); epitopes are schematically illustrated in (**aIII**) (Y)) detect wild-type proteins only in *Dlgap4*^+/+^ but not *Dlgap4*^geo/geo^ animal brains. Immuno-detection of β-tubulin in the corresponding lanes is shown in the panels below. For more details see text. **e** Haematoxylin and eosin stained coronal tissue sections from *Dlgap4*^+/+^ and *Dlgap4*^geo/geo^ brains. Lower panels: magnifications of the hippocampal regions shown above. Scale bar: 1 mm

The BayGenomics embryonic stem cell line XH723 (RRID:CVCL_PY94; breed/subspecies: 129P2/Ola)^29^ was utilized to generate a SAPAP4-deficient (*Dlgap4*^geo/geo^) mouse line that was maintained on a C57BL/6J background (Fig. 1a). At the mutated allele (*Dlgap4*^geo^), integration of an exon trap vector leads to the synthesis of an about 6.5 kb fusion mRNA, which was detected by Northern blotting using total RNA of whole brains, and the loss of 5.0 and 3.8 kb SAPAP4 mRNAs synthesized from the wild-type allele (Fig. 1b). Thus, *Dlgap4*^geo^ encodes an artificial protein (SAPAP4-geo, schematically shown in Fig. 1aIII) that consists of the N-terminal half of SAPAP4 fused to β-galactosidase (β-gal) and neomycin phosphotransferase (neo) and was identified by Western blotting with an anti-β-gal antibody (Fig. 1c, arrowhead). Notably, SAPAP4-geo lacks the known interaction site for Shank family members^1,3,4^ thus disrupting the molecular link between Dlg and Shank proteins. Smaller immuno-reactive bands (indicated by grey arrows in Fig. 1c) appear to represent breakdown products of SAPAP4-geo, thus indicating its increased instability. Noteworthy, further Western blot analyses of PSD proteins with two antibodies recognizing distinct epitopes in the C-terminal half of SAPAP4 (schematically depicted in Fig. 1aIII) showed that wild-type SAPAP4 with an apparent molecular weight of about 145 kDa is not detected in *Dlgap4*^geo/geo^ mouse brains (Fig. 1d). Taken together, these data show that the integration of the gene trap cassette into *Dlgap4* disrupts the synthesis of wild-type SAPAP4 mRNAs and proteins in the *Dlgap4*^geo/geo^ mouse brain.

Heterozygous *Dlgap4*^+/geo^ and homozygous *Dlgap4*^geo/geo^ males and females are viable and fertile. Homozygous *Dlgap4*^geo/geo^ animal frequency for offspring from heterozygous breeding pairs was slightly lower than the expected Mendelian ratio (19% *Dlgap4*^geo/geo^; 55% *Dlgap4*^+/geo^; 26% *Dlgap4*^+/+^; *n* = 450), while the sex distribution was nearly balanced (48% males and 52% females).

### Loss of SAPAP4 affects dendritic arborisation as well as structure and function of excitatory synapses

**Fig. 2:**
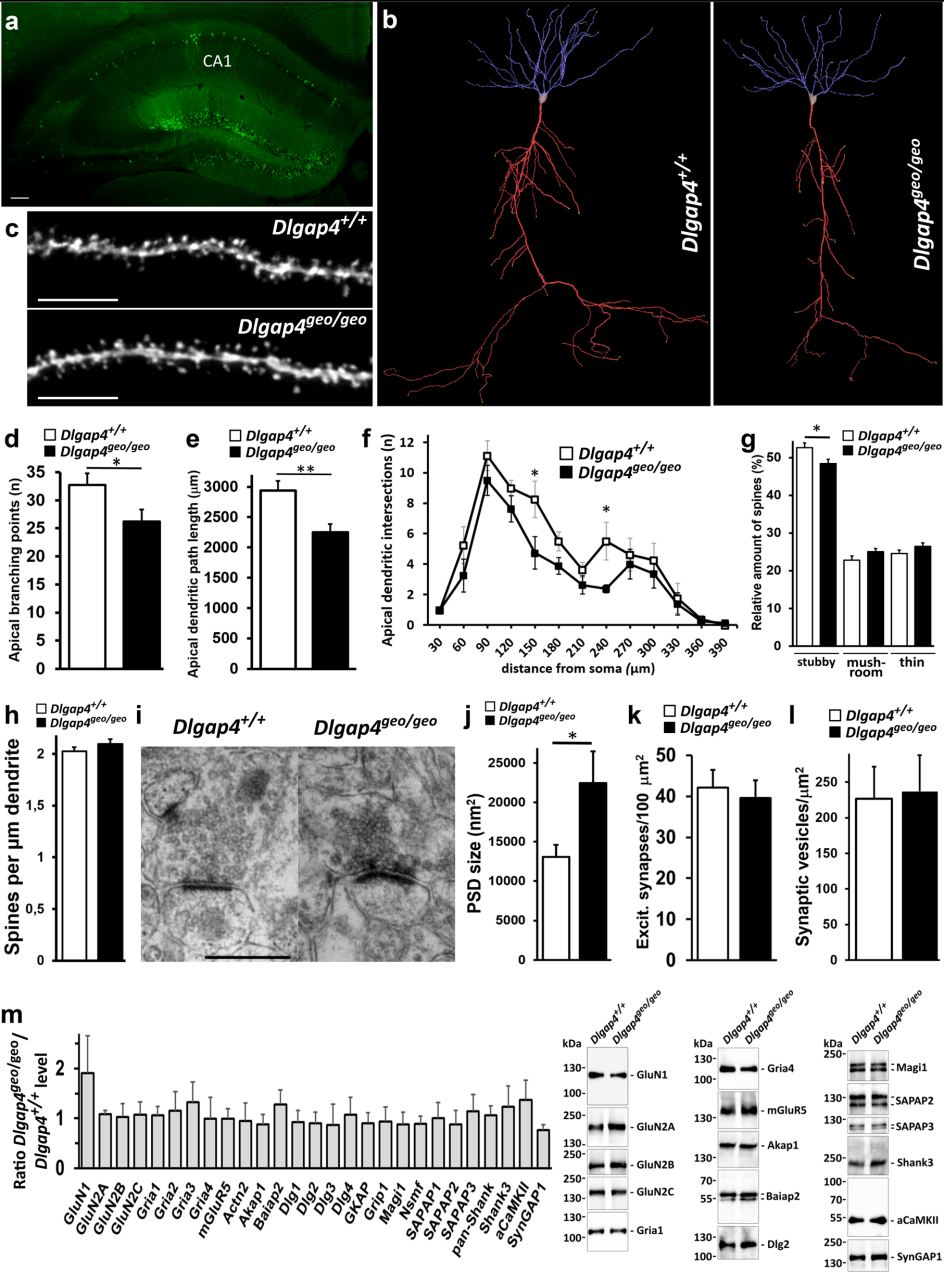
Altered dendritic arborisation, synaptic spines and PSDs of CA1 hippocampal neurons from *Dlgap4*^geo/geo^ mice. **a** Fluorescent micrograph of the hippocampal area obtained from a brain tissue slice of a *Dlgap4*^geo/geo^ mouse showing EGFP labelling of pyramidal and granule neurons (green). The position of the CA1 region is indicated. Scale bar: 100 μm. **b** 3D reconstruction of representative CA1 pyramidal neurons showing complete apical (red) and basal (blue) dendritic trees. **c** Representative images of dendritic segments from hippocampal CA1 pyramidal cells. Scale bar: 10 μm. **d**, **e** Bar graphs showing the mean total number of apical branching points (**d**) and mean total apical dendritic path length (**e**) of CA1 neurons (*p < 0.05, **p < 0.01, Fisher’s exact test; *n* = 8 neurons/genotype). Simple vertical lines represent SEM. **f** Sholl analysis plot showing the mean number of intersections between apical dendrites and a series of concentric spheres centered at the soma of CA1 neurons (*p < 0.05, *t*-test; *n* = 8 neurons/genotype). Simple vertical lines represent SEM. **g**, **h** Bar graphs displaying the fraction of stubby, mushroom-type and thin dendritic spines (**g**) and the spine density (**h**) obtained from EGFP labelled pyramidal neurons of the hippocampal CA1 region of *Dlgap4*^geo/geo^ and *Dlgap4*^+/+^ male mice (14–20 weeks old; *p < 0.05, *t*-test; *n* = 10 animals/genotype, ≥63 dendritic segments/genotype, ≥3670 spines/genotype). Simple vertical lines represent SEM. **i** Representative EM micrographs of excitatory synapses from the hippocampal CA1 area of adult *Dlgap4*^*+/+*^ and *Dlgap4*^geo/geo^ mice. Scale bar: 500 nm. **j**–**l** Bar graphs showing the mean PSD size (**j** *p < 0.05, *t*-test; *n* = 3 mice/genotype, ~70 synapses/animal), the mean density of excitatory synapses (**k** *n* = 3 mice/genotype, ~13,500 μm^2^ CA1 area/mouse) and the mean density of synaptic vesicles (**l** *n* = 3 mice/genotype, 75 presynaptic boutons/genotype) obtained from EM micrographs of the hippocampal CA1 molecular layer of adult male mice. **m** *Dlgap4*^geo/geo^ to *Dlgap4*^+/+^ ratio in the levels of distinct PSD components. In PSD enriched fractions obtained from hippocampal lysates of adult mice, individual proteins were quantified by Western blotting (representative examples are shown on the right). In the bar graph (left), a value of 1 indicates that the level of the respective PSD component is identical in *Dlgap4*^geo/geo^ and *Dlgap4*^+/+^ brain fractions. For each protein, data are based on at least three different PSD preparations and three independent Western blot experiments per preparation. Simple vertical lines in (**j**–**m**) represent SD. The following abbreviations have been used: GluN: glutamate receptor ionotropic, NMDA; Gria: glutamate receptor ionotropic, AMPA; mGluR5: metabotropic glutamate receptor 5; Actn2: alpha-actinin-2; Akap1: A-kinase anchor protein 1, mitochondrial; Baiap2: brain-specific angiogenesis inhibitor 1-associated protein 2; Dlg: disks large homolog; GKAP: guanylate kinase-associated protein; Grip1: glutamate receptor-interacting protein 1; Magi1: magi1 protein; Nsmf: NMDA receptor synaptonuclear signaling and neuronal migration factor; SAPAP: SAP90/PSD-95-associated protein; Shank: SH3 and multiple ankyrin repeat domains protein; αCaMKII: calcium/calmodulin-dependent protein kinase type II subunit alpha; SynGAP1: Ras/Rap GTPase-activating protein SynGAP1. For further details see text

*Dlgap4*^geo/geo^ animals displayed a normal overall appearance and no alterations were observed with respect to all investigated gross morphological parameters. In particular, overall brain structure of *Dlgap4*^geo/geo^ mice was normal (Fig. 1e). To assess whether the loss of SAPAP4 alters dendritic arborisation or the shape/density of dendritic spines, we cross-bred *Dlgap4*^geo/geo^ mice with thy1-GFP line M animals exhibiting sparse enhanced green fluorescent protein (EGFP) labelling of hippocampal pyramidal neurons^30^ (Fig. 2a). Confocal 3D image stacks were used to reconstruct complete EGFP labelled CA1 neurons (Fig. 2b) or dendritic segments (Fig. 2c)^31–33^. Thus, we found that in the brain of *Dlgap4*^geo/geo^ mice both the mean total number of branching points (Fig. 2d) and mean total path length of apical dendrites (Fig. 2e) are significantly decreased compared to *Dlgap4*^+/+^ animals. For basal dendrites, both parameters are slightly but not significantly reduced (mean total number of branching points, *Dlgap4*^geo/geo^: 16.0 ± 2.73, *Dlgap4*^+/+^: 19.13 ± 4.73, *p* < 0.128; mean total path length, *Dlgap4*^geo/geo^: 1711.78 ± 310.96 µm, *Dlgap4*^+/+^: 1989.43 ± 313.26; *p* < 0.097; Fisher’s exact test, *n* = 8 per genotype). In accordance with this finding, Sholl analysis showed a significantly diminished mean number of apical dendritic intersections at 150 and 240 µm distance from the soma in *Dlgap4*^geo/geo^ mice (Fig. 2f), while it did not reveal significant differences for basal dendritic trees (data not shown). Analysis of dendritic spine morphology further revealed a slight but significant decrease in the fraction of stubby spines in *Dlgap4*^geo/geo^ mice compared to *Dlgap4*^+/+^ animals, while the relative amounts of mushroom-type and thin spines increased to some extent but not significantly (Fig. 2g). The mean spine density remained unchanged (Fig. 2h). Synaptic ultrastructure was further analysed by electron microscopy (EM). We sampled micrographs from randomly chosen areas of the hippocampal CA1 *stratum radiatum* of *Dlgap4*^geo/geo^ and *Dlgap4*^+/+^ mice. In general, the basic structure of excitatory synapses in the *Dlgap4*^geo/geo^ brain appeared to be intact. Synapses possessed normally shaped presynaptic boutons containing numerous synaptic vesicles and a dendritic spine containing a clearly visible PSD that was tightly associated with the postsynaptic membrane (Fig. 2i). While the mean area covered by the PSD was increased by 70% in *Dlgap4*^geo/geo^ compared to *Dlgap4*^+/+^ animals (Fig. 2j), the mean density of both excitatory synapses (Fig. 2k) and synaptic vesicles were unchanged (Fig. 2l). The penultimate result is in agreement with our above finding that the mean spine density along dendritic shafts of EGFP labelled pyramidal neurons is identical in *Dlgap4*^geo/geo^ and *Dlgap4*^+/+^ mice (Fig. 2h). To determine if the loss of SAPAP4 may also change the molecular framework of the PSD, we compared the levels of different postsynaptic proteins in PSD enriched fractions derived from hippocampi of *Dlgap4*^geo/geo^ and *Dlgap4*^+/+^ animals. Equal amounts of protein were analysed by Western blotting with antibodies specific for single PSD components. By focussing mainly on scaffold proteins and receptor subunits, we observed that the in vivo loss of SAPAP4 does not noticeably alter the molecular composition of the PSD (Fig. 2m). Noteworthy, we did not observe a compensatory increase in the level of other family members. By trend, postsynaptic levels of the NMDAR subunit GluN1 were found to be enlarged. Taken together, our findings show that SAPAP4 plays an important role in regulating PSD size, while exhibiting only minor effects on spine density and shape.

**Fig. 3:**
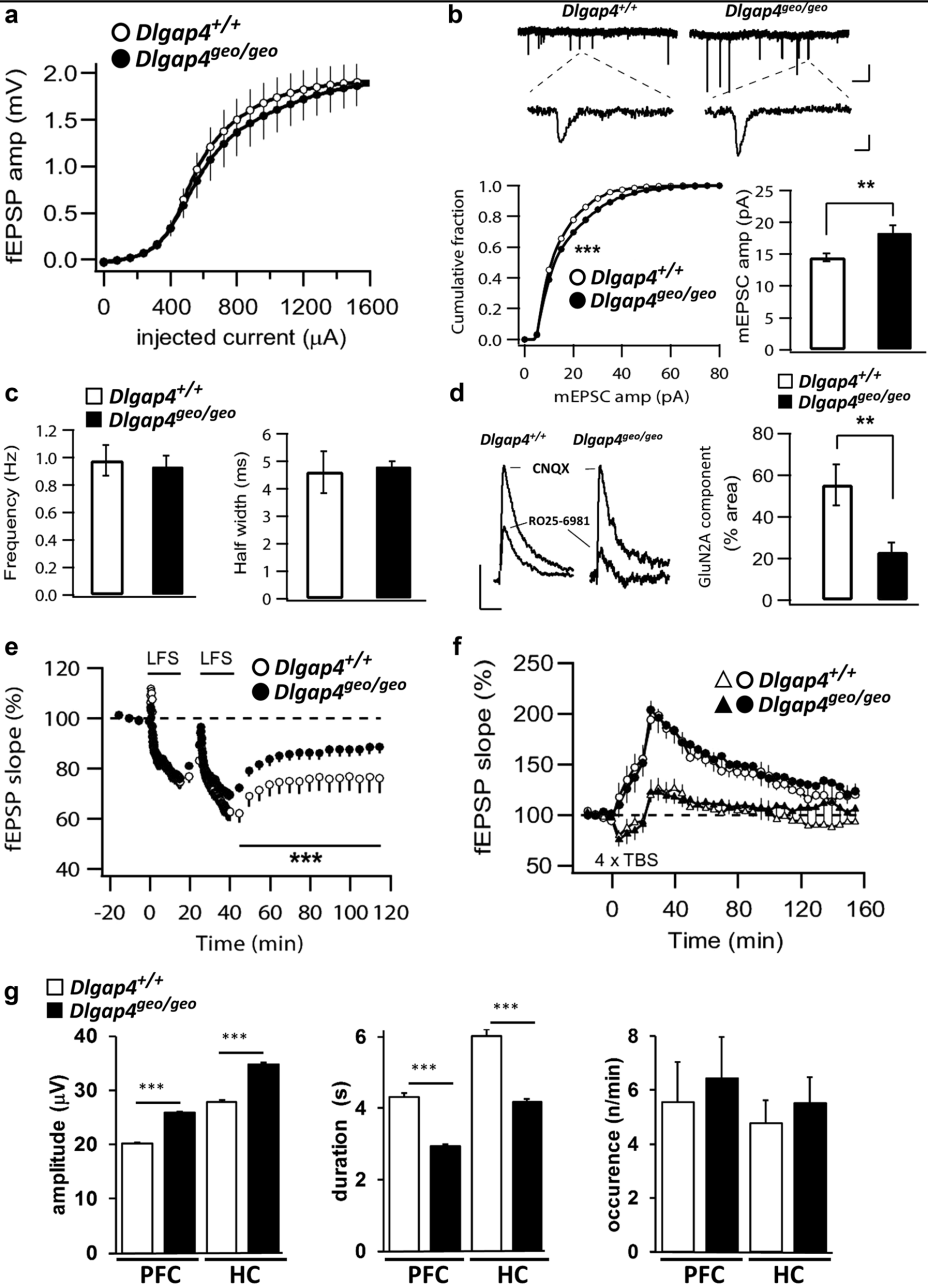
Impaired synaptic transmission, plasticity and network activity in SAPAP4-deficient mouse brains. **a** Input/output curves of fEPSPs in CA1 *stratum radiatum* are indistinguishable between *Dlgap4*^+/+^ and SAPAP4-deficient littermates. **b** AMPAR-mediated mEPSCs are significantly larger in SAPAP4-deficient mice. Upper panel: raw traces recorded in exemplary neurons presented at low (upper traces, scale bars 1 s and 20 pA) and at high resolution (lower traces, bars are 10 ms and 10 pA). Lower left panel: cumulative histogram of mEPSC amplitudes shows a significant right-shift in SAPAP4-deficient neurons (**p < 0.01, 3600 events collected from 12 *Dlgap4*^geo/geo^ cells and 4150 events from 14 *Dlgap4*^+/+^ cells, Kolmogorov–Smirnov 2 sample test). Lower right panel: the mean mEPSC amplitude is significantly enhanced in SAPAP4-deficient neurons (**p < 0.01, *t*-test; *n* = 12 *Dlgap4*^geo/geo^ and 14 *Dlgap4*^+/+^ cells). **c** mEPSC frequency (left bar graph) and half-width (right) were indistinguishable between *Dlgap4*^geo/geo^ and *Dlgap4*^+/+^ neurons. **d** The GluN2A component of the NMDAR-mediated eEPSC is significantly decreased in SAPAP4-deficient neurons (**p < 0.05, *t*-test; *n* = 10 *Dlgap4*^geo/geo^ and 9 *Dlgap4*^+/+^). Left panel: exemplary traces of NMDAR-mediated eEPSCs at +40 mV in the presence of CNQX and after application of the GluN2B-antagonist RO 25-6981. Scale bars: 5 ms and 30 pA for *Dlgap*^+/+^ and 5 ms and 20 pA for *Dlgap*^geo/geo^ eEPSCs. **e** Following LFS, SAPAP4-deficient slices (*n* = 14 slices, 4 mice) exhibit reduced LTD compared with *Dlgap4*^+/+^ slices (*n* = 14 slices, 4 mice). Two-way ANOVA revealed significant effects of genotype (F_1,14_ = 59.2, ***p < 0.001) and time (F_1,14_ = 2.1, *p < 0.05) but no interaction between these factors (F_1,14_ = 0.02, *p* > 0.05). **f** LTP induced by repetitive thetaburst stimulation (4× TBS) is identical in SAPAP4-deficient (*n* = 30 slices, 8 mice) and *Dlgap4*^+/+^ (*n* = 30 slices, 7 mice) animals (circles and triangles represent stimulated and control pathways, respectively). Two-way ANOVA revealed a significant effect of time (F_1,30_ = 7.48, *p < 0.05) but no significant effect of genotype (F_1,30_ = 2.13, *p* > 0.05) or an interaction between them (F_1,30_ = 0.17, *p* > 0.05). Experiments shown in (**a**–**f**) were performed with 12–16-week-old male mice. **g** Discontinuous oscillatory in vivo activity of prefrontal (PFC) and hippocampal (HC) networks of neonatal (P8–12) mice (8 animals/genotype). The bar graphs illustrate that in both investigated brain areas the amplitude of oscillatory events was significantly amplified in *Dlgap4*^geo/geo^ mice (*p* < 0.001, two-sample Kolmogorov–Smirnov test), whereas their duration was shortened (*p* < 0.001). SAPAP4 deficiency did not alter the occurrence of oscillatory events in both brain regions (*p* = 0.99). Simple vertical lines represent SEM. For more details see text

To analyse whether the dramatic increase in average PSD area leads to alterations in synaptic transmission, we performed extracellular field and whole-cell patch-clamp recordings from CA1 pyramidal neurons in acute hippocampal slices. Input/output curves of field excitatory postsynaptic potentials (fEPSPs) were similar between *Dlgap4*^+/+^ and *Dlgap4*^geo/geo^ animals (Fig. 3a). We observed a significant rise in the average amplitude of miniature excitatory postsynaptic currents (mEPSCs) in SAPAP4-deficient compared to *Dlgap4*^+/+^ brains indicating an increased AMPAR response at active synapses (Fig. 3b). However, both average half width and frequency of mEPSCs were not affected by the loss of SAPAP4 (Fig. 3c), showing that the electrophysiological characteristics of synaptic AMPARs are unchanged and that SAPAP4 loss does not overtly affect presynaptic neurotransmitter release. Intracellular recordings of evoked excitatory postsynaptic currents (eEPSCs) utilizing different subtype-specific glutamate receptor inhibitors revealed that in CA1 pyramidal cells the relative amount of GluN2A-mediated NMDAR currents is significantly decreased in *Dlgap4*^geo/geo^ mice (Fig. 3d), indicating that SAPAP4 promotes the early postnatal switch from mostly GluN2B- to more GluN2A-containing NMDAR in the hippocampus^34^. To assess how these changes in synaptic transmission influence the plasticity of synapses, we tested the ability to induce long-term depression (LTD) and long-term potentiation (LTP) at hippocampal CA1 pyramidal cell synapses. While LTD induced by low-frequency stimulation was impaired in *Dlgap4*^geo/geo^ hippocampus (Fig. 3e), LTP induced by repetitive thetaburst stimulation of the Schaffer collaterals remained intact (Fig. 3f). To assess if SAPAP4 deficiency may affect the maturation of global in vivo neuronal network activity, we monitored discontinuous oscillatory activity of neuronal networks in the prefrontal cortex (PFC) and hippocampus by performing multi-site recordings of local field potentials (LFP) and spiking activity from neonatal mice^35,36^. Here, we found that in both brain regions the amplitude of oscillatory events was significantly increased in vivo in *Dlgap4*^geo/geo^ animals compared to *Dlgap4*^+/+^ mice, while there was a significant decline in their duration (Fig. 3g). The occurrence of oscillatory events in both brain areas was unchanged. In summary, these findings indicate that SAPAP4 may play a role in the control of AMPAR-mediated synaptic strength, maturation of excitatory synapses and functioning of neuronal circuits.

### ***Dlgap4***^***geo/geo***^**mice exhibit hyper-locomotion and disrupted novelty-induced exploratory behaviour**

**Fig. 4:**
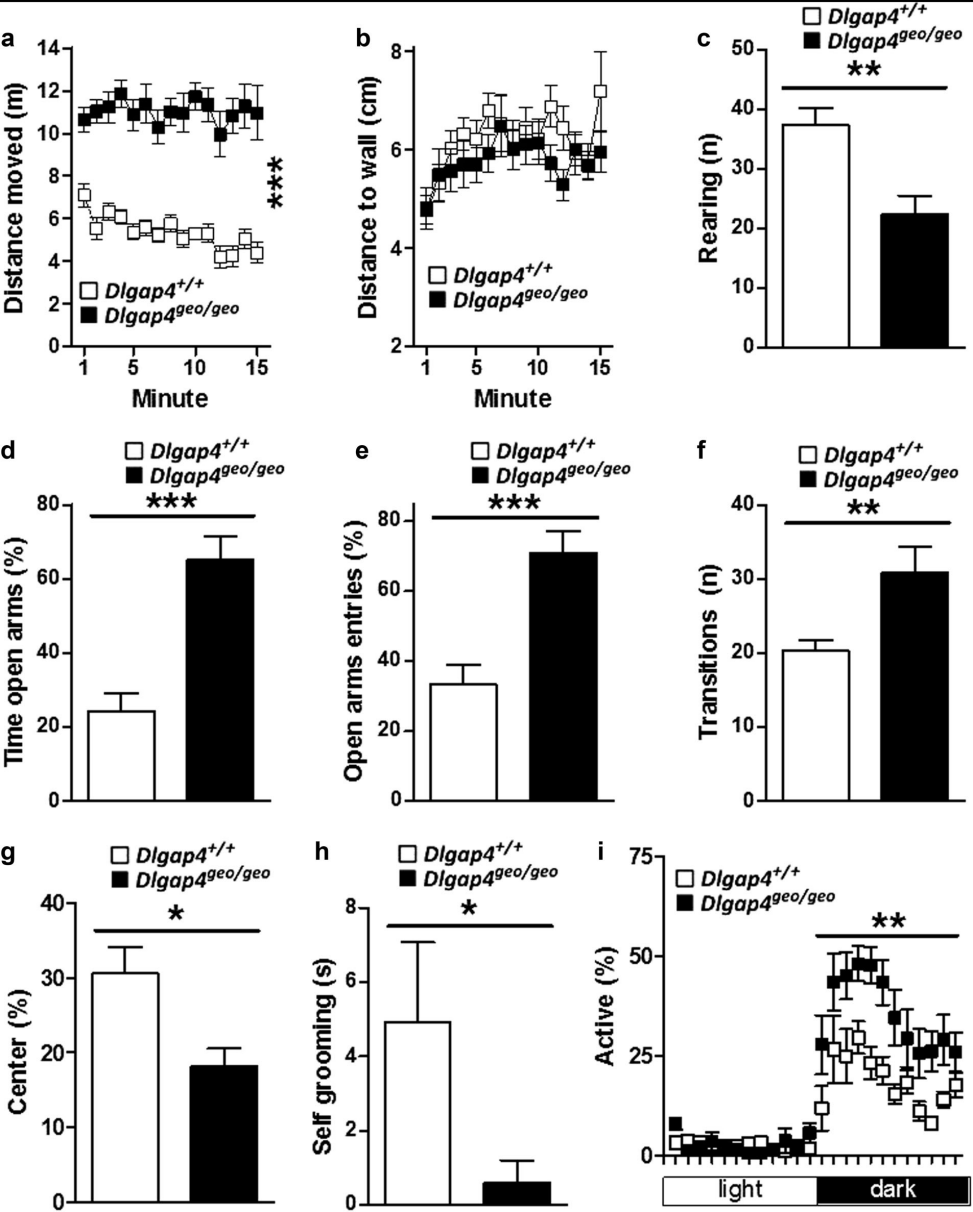
Altered novelty-induced exploratory behaviour in SAPAP4-deficient mice. **a** *Dlgap4*^geo/geo^ mice covered longer distances in the open field test as compared to *Dlgap4*^+/+^ mice (***p < 0.001, effect of genotype after mixed two-way ANOVA). **b** Thigmotactic behaviour in the open field was not affected in *Dlgap4*^geo/geo^ mice. **c** Rearing, a typical novelty-induced exploratory behaviour in mice, was reduced in *Dlgap4*^geo/geo^ mice during the first 5 min of the open field compared to *Dlgap4*^+/+^ mice. **d**–**h** In the elevated plus maze test, *Dlgap4*^geo/geo^ mice spent more time on the open arms (**d**), entered the open arms more often (**e**), did more total transitions (**f**), spent less time on the centre (**g**) and self-groomed less (**h**) compared to *Dlgap4*^+/+^ mice (in (**c–h**): *p < 0.05, **p < 0.01 and ***p < 0.001; unpaired *t*-test). **i** During the dark cycle, *Dlgap4*^geo/geo^ mice in the home cage were more active than *Dlgap4*^+/+^ mice. (**p < 0.01 as compared to *Dlgap4*^+/+^ mice within the time bin, Newman–Keuls post-hoc test after mixed three-way ANOVA). Simple vertical lines represent SEM. All experiments were performed with 11 adult male mice/genotype (cohort 1). For more details see text

As genetic variants of *DLGAP1-4* have been linked to abnormal behaviour in humans^9,12–19^, we performed a variety of behavioural tests with littermates from heterozygous breeding pairs. *Dlgap4* ablation profoundly altered novelty-induced exploratory behaviour as *Dlgap4*^geo/geo^ mice showed hyper-locomotion indicated by the longer distance moved in the open field test (Fig. 4a). Noteworthy, *Dlgap4*^geo/geo^ mice did not display any short-term (‘within-session’) habituation. While the distance moved by *Dlgap*^+/+^ mice diminished as the trial progressed, the mean distance travelled by *Dlgap4*^geo/geo^ mice remained constant (Fig. 4a). Also, the number of rearing bouts, a typical exploratory behaviour in rodents^37^, was significantly reduced in SAPAP4-deficient mice compared to *Dlgap4*^+/+^ littermates (Fig. 4c), whereas thigmotactic behaviour was not affected (Fig. 4b). These observations show that *Dlgap4*^geo/geo^ mice exhibit severe hyper-locomotion and impaired habituation learning, while reduced rearing indicates that novelty-induced exploratory behaviour may be disturbed.

In the elevated plus maze (EPM) test, significantly more *Dlgap4*^geo/geo^ mice fell from the open arm as compared to *Dlgap4*^+/+^ littermates (*Dlgap4*^geo/geo^: 5/11, *Dlgap4*^+/+^: 0/11; *p* < 0.05, Fisher’s exact test). Also, ambulation of *Dlgap4*^geo/geo^ animals lacked the typical ‘flat’ posture displayed by mice walking on an elevated open arm for the first time (data not shown). Both observations imply that *Dlgap4*^geo/geo^ mice display impaired risk-assessment, lack of attention, sensory-motor deficits, or a combination of these factors. Behavioural analysis of all mice that completed the whole 5 min EPM test phase (*n* = 6 *Dlgap4*^geo/geo^ and 11 *Dlgap4*^+/+^ males) revealed a high preference of *Dlgap4*^geo/geo^ animals for exploring open versus closed arms (Fig. 4d, e), indicative of a decrease in anxiety. Also, *Dlgap4*^geo/geo^ mice displayed an enhanced general locomotion as indicated by the increased number of transitions during the test period (Fig. 4f), consistent with the hyperactivity displayed in the open field test. Furthermore, *Dlgap4*^geo/geo^ animals spent less time in the centre of the maze (Fig. 4g), a position in which mice normally remain to assess potential risks of entering the open arms, thus again indicating a disrupted risk-assessment. Finally, similar to the open field test the number of rearing bouts was reduced (*Dlgap4*^geo/geo^: 3.0 ± 1.3, *Dlgap4*^+/+^: 14.5 ± 1.1; *p* < 0.001, Fisher’s exact test), further supporting an impaired novelty-induced exploratory behaviour. Given the OCD-like behaviour of *Dlgap3*^−/−^ mice^23^, we also monitored self-grooming. Up to 1-year-old *Dlgap4*^geo/geo^ animals did not display any signs of skin lesions in head, neck or snout regions that may have arisen from excessive self-grooming (data not shown). During the EPM test phase, self-grooming was only displayed by one out of six tested *Dlgap4*^geo/geo^ mice. As all tested *Dlgap4*^+/+^ littermates (*n* = 11) engaged in self-grooming, its total time was significantly reduced in *Dlgap4*^geo/geo^ animals (Fig. 4h; *p* < 0.01, Fisher’s exact test). Thus, *Dlgap4*^geo/geo^ mice spent less time in a posture indicative of a conflictual state and arousal^38^.

When kept in their home cages, *Dlgap4*^geo/geo^ mice displayed an increased locomotor activity during the dark phase (Fig. 4i; *p* < 0.01, Newman–Keuls post-hoc test) that was similar to the enhanced activity observed in the open field and EPM test. The general circadian activity profile however was unaltered. Thus, hyper-locomotion of *Dlgap4*^geo/geo^ mice affects both home cage activity and adaptive behavioural responses.

### **Cognitive functioning is disrupted in*Dlgap4***^***geo/geo***^**mice**

**Fig. 5:**
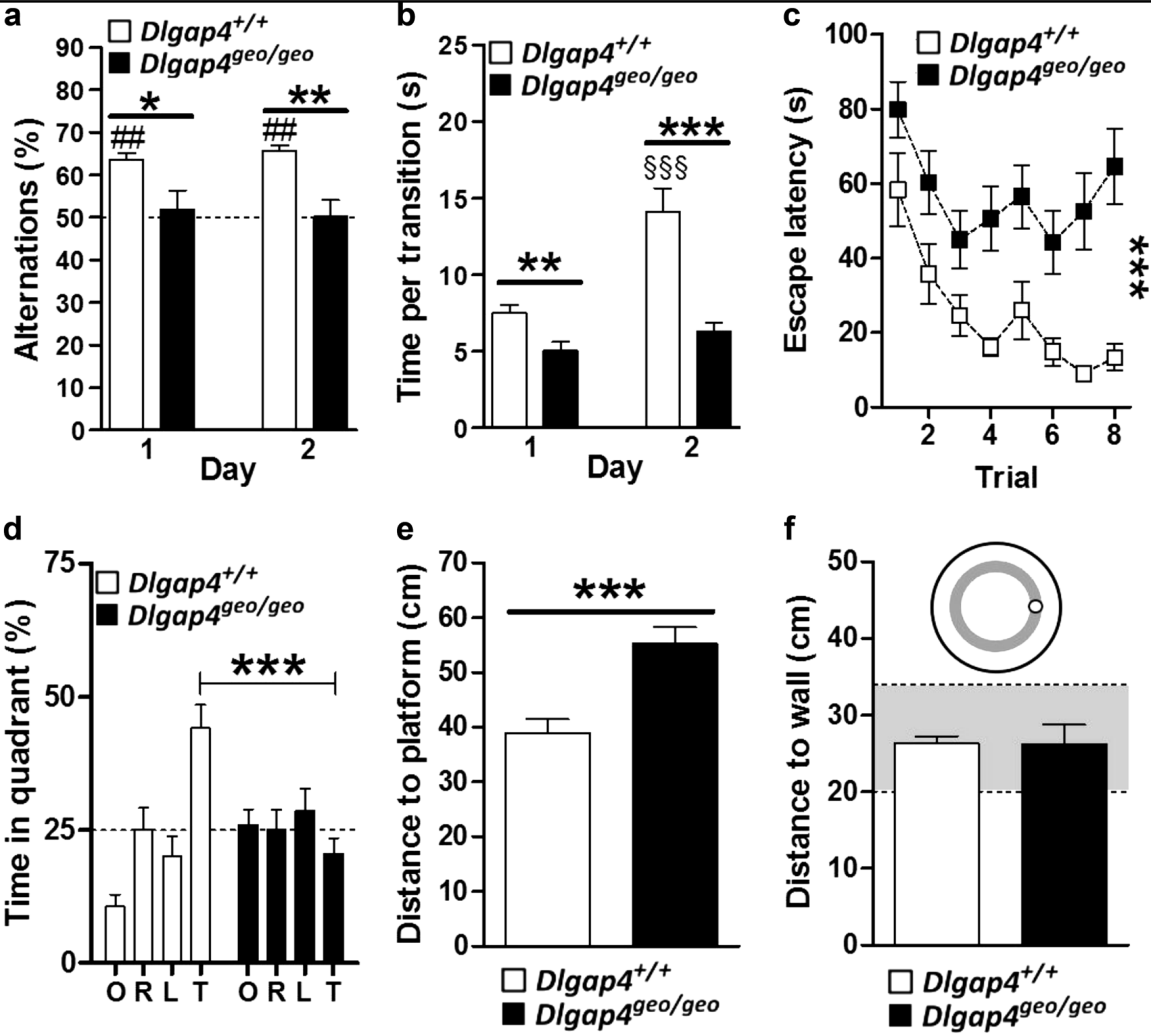
Impaired cognitive functioning in SAPAP4-deficient mice. **a** During both days of the spontaneous alternation test, *Dlgap4*^+/+^ mice exhibited higher percentages of alternations than expected by chance (50%, indicated by dotted line; ^##^*p* < 0.01, Wilcoxon signed rank test) and as compared to *Dlgap4*^geo/geo^ mice (*p < 0.5 and **p < 0.01, Mann–Whitney test). **b** The average time to perform a transition increased from day 1 and day 2 in *Dlgap4*^+/+^, but not *Dlgap4*^geo/geo^ mice (^§§§^*p* < 0.001 Newman–Keuls post-hoc test after mixed two-way ANOVA) and was lower in *Dlgap4*^geo/geo^ compared to *Dlgap4*^+/+^ mice (**p < 0.01 and ***p < 0.001, Newman–Keuls post-hoc test after mixed two-way ANOVA). **c** Impaired spatial learning in *Dlgap4*^geo/geo^ mice as indicated by the increased escape latencies during the learning trials of the water maze test (***p < 0.001, effect of genotype after mixed two-way ANOVA). **d–f** During the probe trial of the water maze test, *Dlgap4*^geo/geo^ mice spent less time in the target quadrant (**d** ***p < 0.001, Newman–Keuls post-hoc test after mixed two-way ANOVA) and swam at longer distances from the platform compared to *Dlgap4*^+/+^ mice (**e** ***p < 0.001, unpaired *t*-test). However, mice of both genotypes swam at the appropriate distance from the maze wall in order to find the platform (**f** the entire area with a platform to wall distance identical to the respective distance during the learning trials is displayed in grey). Simple vertical lines represent SEM. All experiments were performed with 11 adult male mice/genotype (cohort 1). For more details see text

To assess cognitive function, we performed the spontaneous alternation test. It relies on the preference of mice to rather explore unfamiliar than familiar stimuli. Given the choice to enter two different arms of a Y-shaped maze, freely moving animals will thus choose to enter the less recently visited arm. To successfully perform this task, animals need to remember the order in which the three arms of the maze have been visited, a function requiring working memory. While *Dlgap4*^+/+^ mice showed the expected preference for the less recently visited arm during both sessions performed on days 1 and 2 (Fig. 5a), *Dlgap4*^geo/geo^ mice alternated at chance level (50%) potentially suggesting working memory impairment. However, this poor performance may as well result from their hyperactive behaviour and a consequential decreased attention, an assumption that is supported by the fact that *Dlgap4*^geo/geo^ mice moved faster between arms than *Dlgap4*^+/+^ animals (Fig. 5b). Thus, we investigated spatial learning and memory in the water maze test, an experiment that does not involve novelty-seeking as the spontaneous alternation test. Spatial learning and memory was significantly impaired in *Dlgap4*^geo/geo^ mice as indicated by their poorer performance during learning (Fig. 5c) as well as by their searching strategy during the probe trial (Fig. 5d–f). During learning, they required more time to find the hidden platform (Fig. 5c) compared to *Dlgap4*^+/+^ mice, despite a similar swim velocity (data not shown). During the probe trial, *Dlgap4*^geo/geo^ mice spent equal amounts of time in all four quadrants with no preference for the target quadrant (Fig. 5d). Also, they searched at larger mean distance to the former platform position compared to *Dlgap4*^+/+^ mice (Fig. 5e). These data indicate that *Dlgap4*^geo/geo^ mice were not able to locate the hidden platform and most likely only found it based on an alternative searching strategy such as circling. Therefore, SAPAP4-deficient animals use simple information as for example the approximate distance of the platform from the pool wall (Fig. 5f). Taken together, data obtained with the spontaneous alternation and water maze test suggest that SAPAP4 deficiency leads to cognitive deficiencies resulting from impaired spatial learning, attention deficits or a combination of both.

### ***Dlgap4***^***geo/geo***^**mice exhibit impaired social behaviour and vocal communication**

**Fig. 6:**
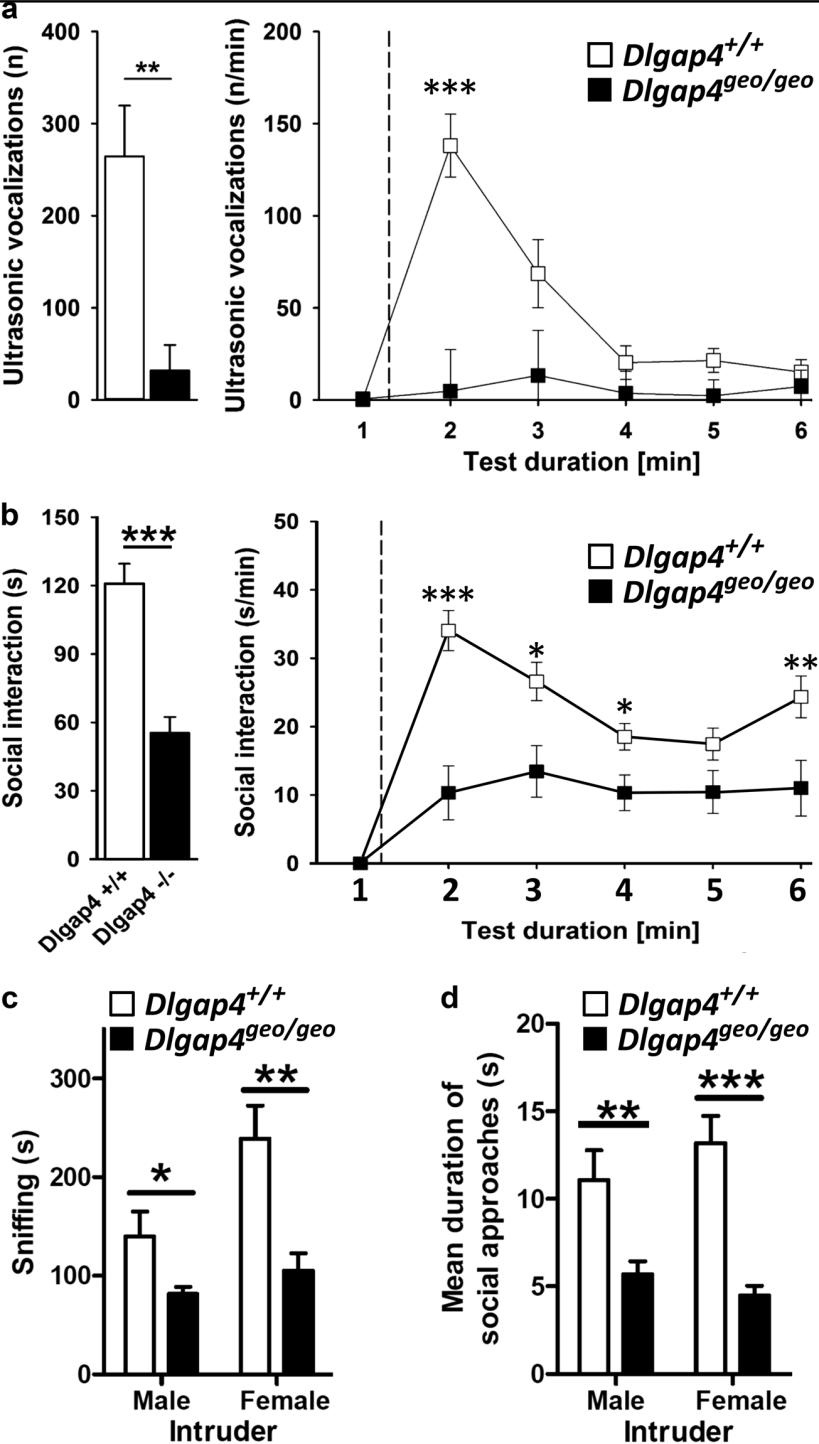
Impaired social behaviour and vocal communication in SAPAP4-deficient mice. **a** Total number of ultrasonic vocalizations emitted during the 5 min social interaction test in juveniles (bar graph), with the time course for the number of ultrasonic vocalizations emitted for each 1 min time bin across the 5 min social interaction period, plus 1 min habituation (line diagram; dashed line indicates introduction of partner mouse; repeated measurements ANOVA with the between-subject factor genotype and the between-subject factor test duration; **p < 0.01 and ***p < 0.001). **b** Total social interaction time displayed during the 5 min direct reciprocal social interaction test in juveniles (bar graph), with the time course for the social interaction time displayed during each 1 min time bin across the 5 min social interaction period, plus 1 min habituation (line diagram; dashed line indicates introduction of partner mouse; repeated measurements ANOVA with the between-subject factor genotype and the between-subject factor test duration; *p < 0.05, **p < 0.01, ***p < 0.001). Experiments shown in (**a** and **b**) were performed with 18 *Dlgap4*^+/+^ (9 male and 9 female) and 11 *Dlgap4*^geo/geo^ mouse pairs (6 male and 5 female) from cohort 2, respectively. **c**, **d** In adulthood, *Dlgap4*^geo/geo^ mice spent less time investigating a male or female conspecific during direct reciprocal social interaction (**c**). On average, each social approach was significantly shorter as compared to *Dlgap4*^+/+^ mice (**d**) (*p < 0.05, **p < 0.01, ***p < 0.001; Newman–Keuls post-hoc test after mixed two-way ANOVA). Simple vertical lines represent SEM. Experiments shown in (**c** and **d**) were performed with 10 adult male focal mice/genotype (cohort 3). For more details see text

In humans, *DLGAP1-4* have been identified as risk genes for different neuropsychiatric disorders^39,40^. Thus, we tested how *Dlgap4*^geo/geo^ mice respond to social stimuli. To assess communicative functions, we recorded ultrasonic vocalizations (USVs) of juvenile mice emitted during direct reciprocal social interaction in same-genotype pairs. In this situation, USVs appear to have a pro-social affiliative function and may help to maintain social proximity^41^. The total number of USVs emitted throughout the entire test session was dramatically reduced in *Dlgap4*^geo/geo^ mice compared to *Dlgap4*^+/+^ animals (Fig. 6a). This was accompanied by a strong decrease in the time *Dlgap4*^geo/geo^ mice engaged in social interactions (Fig. 6b). The reduction in both parameters was particularly apparent during the first minute of the encounter, when *Dlgap4*^+/+^ mice displayed a high interaction rate that gradually faded during the course of the test session.

The above social interaction assay requires the use of same-genotype/-sex pairs as recorded USVs cannot be attributed to an individual mouse. Thus, we next explored direct reciprocal social interactions between adult *Dlgap4*^*geo/geo*^ and *Dlgap4*^+/+^ mice. Here, an unfamiliar age- and body-weight-matched male mouse (intruder) was introduced for 10 min in the home cage of the male focal animal (resident). As no aggressive interaction was observed between residents and intruders, our behavioural analysis was restricted to the following parameters: time spent sniffing the intruder, allo-grooming, digging and self-grooming. Whereas no differences between genotypes were detected for allo-grooming, digging and self-grooming, *Dlgap4*^geo/geo^ mice spent significant less time sniffing the intruder compared to *Dlgap4*^+/+^ mice (Fig. 6c), which was not due to a lower amount of approaches, but to the fact that on average each approach lasted longer in *Dlgap4*^+/+^ mice compared to *Dlgap4*^geo/geo^ mice (Fig. 6d). These findings indicate that SAPAP4-deficient mice have a reduced ability for maintaining social interactions. Similar results were obtained when we used an unfamiliar adult female mouse as intruder (Fig. 6c, d). Thus, *Dlgap4*^geo/geo^ mice display severe deficits in social interaction independent of whether they encounter SAPAP4-deficient or *Dlgap4*^+/+^ mice.

## Discussion

SAPAP4-deficient excitatory CA3–CA1 synapses exhibit several interesting phenotypes, including larger PSDs, elevated levels of AMPAR-mediated currents and an increased GluN2B/GluN2A ratio, changes coinciding with a reduced LTD and an unaltered LTP. Most of these findings are distinct or even contrary to those reported for SAPAP2-deficient neocortical and SAPAP3-deficient cortico-striatal synapses. For example, the loss of either SAPAP2 or SAPAP3 leads to a reduction of both PSD size and AMPAR-based transmission^23,26^. While these data may suggest a synaptic function of SAPAP4 that is distinct from that of SAPAP2 and SAPAP3, they may as well reflect regional differences within the brain, such as varying expression levels of other PSD components.

Functional analysis of hippocampal synapses lacking wild-type SAPAP4 revealed a significantly higher GluN2B/GluN2A ratio. GluN2A and GluN2B are the two predominant GluN2 subunits of NMDARs in the hippocampus^42^. While GluN2B levels are high at birth and decrease into adulthood, GluN2A expression increases with age. Molecular mechanisms mediating this developmental switch are poorly understood^34,42^. The increased GluN2B/GluN2A ratio at SAPAP4-deficient synapses suggests that SAPAP4 is critically involved in molecular events underlying NMDAR subunit transition and maturation of excitatory synapses. Intriguingly, the loss of SAPAP3 at striatal synapses also increases GluN2B/GluN2A ratios^23^, indicating that both proteins promote a similar developmental switch in GluN2 subunit dominance at different synapses. At hippocampal excitatory synapses, NMDARs act as mediators of plasticity^34^. In particular, ‘juvenile’ GluN2B-containing receptors have been suggested to be vital for keeping synapses bidirectionally flexible. Our findings however do not support this hypothesis, as increased GluN2B/GluN2A ratios observed at SAPAP4-deficient synapses coincide with an unaltered LTP and an even reduced LTD. Compared to GluN2A containing receptors, GluN1/GluN2B receptors appear to support a greater Ca^2+^ influx per unit of current^43^. Also, GluN2B tethers calcium/calmodulin-dependent kinase II (CaMKII) to PSDs^44^, thus mediating tight coupling between Ca^2+^ influx and CaMKII-dependent phosphorylation events, such as phosphorylation of Ser831 in GluA1 promoting AMPAR insertion into postsynaptic membranes^45^. Thus, the observed increase in AMPAR activity at SAPAP4-deficient synapses may be a consequence of a larger GluN2B/GluN2A ratio.

*Dlgap4*^geo/geo^ mice displayed profound changes in behaviour and cognition. Most obviously, they exhibited hyper-locomotion combined with impaired habituation learning and reduced novelty-induced exploratory behaviour. Furthermore, they showed deficits in either risk-assessment, attention or sensory-motor control or a mixture of these features. None of these phenotypic alterations has been reported for mice lacking SAPAP1, 2 or 3^23,26,27^. Enhanced locomotion of *Dlgap4*^geo/geo^ animals was not limited to situations, in which mice were exposed to new external stimuli, but was also observed when they were kept in their home cages. Despite this manifest hyperactivity the general circadian activity profile remained normal. In contrast, SAPAP3-deficient mice that are not hyperactive display a disrupted sleep pattern^23^. Also, while SAPAP4-deficient animals spent less time self-grooming, *Dlgap3*^−/−^ mice show excessive levels of self-grooming, a phenotype reminiscent of OCD in humans^23^. Lastly, anxiety levels appear to be diminished in SAPAP4-deficient mice, whereas they are increased in *Dlgap3*^−/−^ animals. These differences appear to be mainly attributable to the fact that *Dlgap3* is the sole family member with high expression levels in the striatum^20,21,23^.

*Dlgap4*^geo/geo^ mice also displayed cognitive impairment. SAPAP4 loss disrupted habituation learning and working memory as indicated by results from the open field and spontaneous alternation test, respectively. Data of the water maze assay further showed that spatial learning and memory capabilities of *Dlgap4*^geo/geo^ animals are strongly compromised. In contrast, spatial learning of *Dlgap2*^−/−^ mice appears to be normal^26^, indicating that both proteins are of varying importance for cellular processes underlying learning and memory.

Finally, our analyses revealed severe social deficits in *Dlgap4*^geo/geo^ mice. During direct reciprocal social interaction, juvenile SAPAP4-deficient mice spend less time on social contacts. Also, the number of emitted USVs, which appear to serve a pro-social affiliative function helping to maintain social proximity^41^, was drastically reduced. Social deficits apparent in juvenile *Dlgap4*^geo/geo^ mice appear to persist into adulthood. In particular, in a direct reciprocal social interaction assay, adult SAPAP4-deficient animals displayed a diminished capability to maintain social contact with unfamiliar *Dlgap4*^+/+^ mice. Noteworthy, *Dlgap1*^−/−^ mice also displayed reductions in sociability^27^. In contrast, *Dlgap2*^−/−^ animals exhibited an enhanced social approach behaviour^26^, a phenotype that at first glance appears to be contrary to social deficits of *Dlgap4*^geo/geo^ and *Dlgap1*^−/−^ mice. Yet, additional tests revealed that the altered approach behaviour of *Dlgap2*^−/−^ animals rather reflects exacerbated aggressiveness. In contrast, adult *Dlgap4*^geo/geo^ mice displayed normal aggression levels in our reciprocal social interaction test. *Dlgap2*^−/−^ mice also exhibited deficits in reversal learning^26^. In humans, this phenotype indicates decreased cognitive flexibility that is a reduced capacity to adjust one’s thoughts or actions in response to situational changes, a phenotype that has been connected to highly perseverative and inflexible behaviours of ASD subjects. Thus, mice deficient for SAPAP1, 2 or 4 display behavioural aberrations reminiscent of ASD-associated social abnormalities in humans^46–48^.

In neonatal *Dlgap4*^geo/geo^ mice, we identified an impairment of in vivo oscillatory events in PFC and hippocampus. In accordance with these findings, a growing body of data indicates that ASD is often associated with alterations in neuronal circuit activity^49–52^. Imbalanced functional coupling of PFC and hippocampus is thought to contribute to socio-emotional ASD phenotypes^53^. Similar to *Dlgap4*^geo/geo^ mice, animals deficient for Shanks or Neuroligins also display an ASD-like social withdrawal phenotype^54–63^. Also, in humans, mutations in *SHANK1-3* and *NLGN1-4* have been linked with neuropsychiatric disorders, in particular ASD^2,11,64,65^. While the molecular underpinnings of ASD-associated behaviour are poorly understood, current evidence suggests that imbalances in the levels of different PSD components disrupt the assembly, functionality and/or maturation of postsynaptic signalling complexes in a similar manner. In agreement with this hypothesis, the SAPAP4-geo fusion protein encoded by the *Dlgap4*^geo^ allele appears to be rather unstable. Moreover, it is lacking the known interaction site for Shank family members. In the future, it will be interesting to test if *Dlgap4*^geo^ is equivalent to a *Dlgap4* null allele or rather represents a hypomorphic allele encoding a SAPAP4-geo fusion protein that retains some biological activity.

So far, it is unclear whether the functional loss of SAPAP4 leads to a similar phenotype in humans and mice. Exome Aggregation Consortium sequencing data suggest that *DLGAP4* is extremely intolerant to even heterozygous loss-of-function (LoF) mutations (pLI score: 0.99). This is probably not due to embryonic lethality as both *Dlgap4*^*+/geo*^ and *Dlgap4*^*geo/geo*^ mice are viable. Recently, a balanced chromosomal translocation t(8;20)(p12;q11.23) in humans was found to cause autosomal dominant, early-onset, non-progressive, mild cerebellar ataxia^66^. This mutation physically disrupts *DLGAP4* on chromosome 20. While chromosome derivative (der)20 carries the most upstream known *DLGAP4* promotor, the complete coding region is translocated to der8. It is not known if the phenotype of heterozygous translocation carriers, which is quite distinct from the phenotype of *Dlgap4*^geo/geo^ mice, is due to genetic alterations associated with der20, der8 or both. Given the apparent intolerance of *DLGAP4* to heterozygous LoF mutations it seems likely that the mild ataxia does not result from *DLGAP4* haploinsufficiency, but is due to an increased monoallelic expression of specific SAPAP4 mRNAs from the locus located on der8^66^.

## Materials and methods

### Generation of mouse lines, genotyping and morphological analyses

Embryonic stem cells (ES; BayGenomics consortium, clone XH723) were used to generate SAPAP4-deficient mice. ES injection into blastocysts and embryo implantation into pseudo-pregnant females were performed by the Transgenic Service Unit at the ZMNH utilizing standard procedures. Chimeric animals were crossbred with C57BL/6J mice to generate heterozygous *Dlgap4*^+/geo^ animals. Mutant mice were back-crossed with C57BL/6J animals for more than ten generations before experiments presented in this study were performed. All mice were raised in the animal facility of the University Medical Centre Hamburg-Eppendorf. Animals were kept under a 12 h/12h-light/dark cycle with food and water ad libitum. All mouse experiments were approved by and conducted in accordance with the guidelines of the Animal Welfare Committee of the University Medical Centre Hamburg-Eppendorf and the ‘Amt für Gesundheit und Verbraucherschutz’ (79/03, 79/11, 105/12, 11/14, ORG491). For genotyping, mouse-tail biopsies were lysed utilizing DirectPCR Lysis Reagent (peqlab, Erlangen, Germany). PCR genotyping was performed using allele-specific primer pairs; *Dlgap4*^+/+^ allele: 5′-CCCTCCCTTCCGTCTGTCCGT-3′/5′-CTAGAGTGACTTCCCGTGTGCAG-3′ (400 bp PCR product) and 5′-CCCTCCCTTCCGTCTGTCCGT-3/5′-GACTTCCCGTGTGCAGATAGACTT-3′ (393 bp); *Dlgap4*^geo/geo^ allele: 5′-CCCTCCCTTCCGTCTGTCCGT-3/5′-CCTCCTTCCCCGGGCAAGGC-3′ (380 bp) and 5′-CCCGTCGTTTTACAACGTCGTGAC-3′/5′-TGAAACGCCGAGTTAACGCCATC-3′ (431 bp).

Pathological examinations were performed as described^67,68^. For the analysis of dendritic arborisation and spine morphology/density, *Dlgap4*^geo/geo^ mice were crossed with thy1-EGFP line M animals^30^ to finally generate *Dlgap4*^+/+^ and *Dlgap4*^geo/geo^ mice with sparse labelling of hippocampal pyramidal neurons with EGFP. To analyse dendritic arborisation, confocal microscopy of complete CA1 neurons was performed with a Zeiss LSM 510 Meta NLO microscope using 488 nm laser excitation^31^. Each neuron was visualised in 8–11 overlapping image tile stacks with a voxel size of 0.3 × 0.3 × 0.45 μm in *x*–*y*–*z* directions. 3D reconstruction of whole neurons was performed using Neuromantic software (University of Reading, Reading, UK) in semi-automated mode. For dendritic spine analysis, confocal imaging of labelled neurons and image analysis were performed as described^32,33^. In short, confocal images of at least 20 μm long apical dendritic segments from hippocampal subfield CA1 (voxel size: 0.08 × 0.08 × 0.20 μm in *x*–*y*–*z* direction) were obtained with an Eclipse TE2000-U microscope (Nikon Instruments, Amsterdam, The Netherlands; 60× oil objective (NA: 1.4), 488 nm argon laser). Image stacks (Nikon.ids files) were processed using *AutoQuant X3* deconvolution software (Media Cybernetics, Duxford, UK). Spine analysis was performed using *3DMA-Neuron* software offering an algorithm-based, semi-automated evaluation of spine morphology in 3D^69^. Statistical evaluation was performed using *t*-test. Electron microscopic analysis was essentially performed as described^70^. Pictures were taken at 20.000× magnification from randomly chosen areas of hippocampal CA1 *stratum radiatum*. Only synapses possessing both synaptic vesicles and a clearly visible PSD were used to determine PSD size and synaptic vesicle density. *ImageJ* software was utilized for area measurements.

### Antibodies, Western and Northern blotting

PSD enriched fractions were prepared as described^71^, snap-frozen in liquid nitrogen and stored at −80 °C. Western blot analysis and utilized antibodies have been described^72^. Following additional antibodies were used: anti-SAPAP4 (HPA056378, Sigma-Aldrich Chemie GmbH, Taufkirchen, Germany), anti-SAPAP4 (HPA054105, Sigma-Aldrich Chemie GmbH, Taufkirchen, Germany), anti-β-tubulin (ab7291, Abcam, Cambridge, UK) and anti-β-galactosidase (Z3783, Promega GmbH, Mannheim, Germany). Immune complexes on membranes were visualized with ECL substrate (ThermoFisher Scientific, Pinneberg, Germany) and the ChemiDoc XRS+ System (Bio-Rad Laboratories, Munich, Germany). The intensity of individual, non-saturated luminescence signals was determined using *ImageLab* software (Bio-Rad Laboratories, Munich, Germany). For Northern analysis, total mouse brain RNA was isolated using TRIzol reagent (ThermoFisher Scientific, Pinneberg, Germany) and blots were generated as described^73^. A cDNA fragment containing nucleotides 423–1186 of rat SAPAP4 transcripts (NM_173145) was labelled with ^32^P-containing nucleotides (Rediprime II labelling system; GE Healthcare, Solingen, Germany) and used to probe blotted total RNAs. Labelled bands were visualized using X-ray films (Kodak GmbH, Stuttgart, Germany).

### In vitro electrophysiology

Mice, 3–4 months old, were anesthetized with isoflurane and decapitated. Brains were rapidly removed and immersed in ice-cold artificial cerebrospinal fluid (ACSF), continuously gassed with a mixture of 95% O_2_, 5% CO_2_. 350 µm transversal slices were cut (Microm 650H vibratome, ThermoFisher Scientific, Pinneberg, Germany) and allowed to recover for 2–4 h in oxygenated warmed ACSF. LTP and LTD experiments were performed in submersion chambers (SyncroBrain, Lohres Research Lohmann Research Equipment, Dortmund, Germany), continuously perfused with warmed, oxygenated ACSF (3.5 ml/min). Two stimulating (concentric stainless steel, 0.1 MΩ impedance) and one recording (platinum/tungsten impedance 0.5–0.8 MΩ, Thomas Recording, Gießen, Germany) electrodes were positioned in hippocampal CA1 *stratum radiatum*. For LTP experiments, ACSF used during slicing, recovery and recordings contained 119 mM NaCl, 2.5 mM KCl, 2.5 mM CaCl_2_, 1.3 mM MgSO_4_, 1.25 mM NaH_2_PO_4_, 26 mM NaHCO_3_, 10 mM glucose and was maintained at 37 °C and pH 7.4. For LTD experiments, ACSF used during slicing, recovery and recordings contained 4.4 mM KCl, 2.0 mM CaCl_2_, 1.0 mM MgSO_4_, 1.25 mM NaH_2_PO_4_, 25 mM NaHCO_3_, 10 mM glucose and was maintained at 30 °C and pH 7.2. fEPSPs were evoked at CA3/Schaffer collateral-CA1 synapses applying a square bipolar pulse (200 ms duration, total) to both electrodes alternately. Input/output (I/O) curves were generated consecutively increasing stimulation intensity from 100 to 1600 µA with 100 µA steps. Stimulation intensity was adjusted to evoke 50% and 70% of the maximum fEPSP amplitude in LTP and LTD experiments, respectively. Baseline and post-stimulus fEPSPs were evoked every 30 s. LTP was induced by repetitive thetaburst stimulation (10 trains of 5 stimuli, intra-train frequency: 200 Hz, inter-train interval: 200 ms). LTD was induced by two episodes of low-frequency stimulation (LFS; 900 pulses each at 1 Hz, episode interval: 10 min). Signals were amplified 1000×, sampled at 10 kHz and filtered at 1 kHz. For each experiment, the fEPSP slope was measured, averaged and normalized to baseline. Across experiments, normalized fEPSPs were averaged and plotted ± standard error of the mean (SEM). For I/O curves, fEPSP amplitudes in each pathway were measured and fitted with a sigmoidal function. Fitted curves were averaged across different experiments and plotted as mean ± SEM. In all experiments, a minimum of four animals per group was used. mEPSCs were measured in voltage clamp from whole cell patch clamped CA1 pyramidal neurons^74^. Inhibitory synaptic transmission was blocked with 2 µM Gabazine (Sigma-Aldrich Chemie GmbH, Taufkirchen, Germany).

To measure GluN2A-mediated synaptic currents, slices were perfused with LTP-ACSF containing 0.05 mM Mg^2+^ after an incision between CA1 and CA3 was performed. Extracellular stimulation in CA1 *stratum radiatum* elicited eEPSCs at a rate of 0.1 Hz. Gabazine (2 µM) and CNQX (50 µM) were added and the cell membrane potential was clamped to +40 mV to isolate the NMDAR eEPSC. GluN2B mediated currents were blocked by RO 25-6981 (2 µM, Sigma-Aldrich Chemie GmbH, Taufkirchen, Germany). The remaining GluN2A current was integrated and divided by the area of the total NMDAR-eEPSC. Data are presented as mean percent ± SEM.

### In vivo electrophysiological analysis

In vivo oscillatory entrainment of prefrontal–hippocampal network activities was assessed by multi-site recordings of LFP and spiking activity from postnatal (P8–12) urethane-anesthetized mice^35,36^. Shortly, pups were anesthetized with isoflurane (induction 5% in O_2_) followed by i.p. administration of urethane (1 g/kg body weight). Fluorescently-labelled recording electrodes (NeuroNexus, Ann Arbor, MI) were inserted into the prelimbic subdivision of the PFC (PL, perpendicular to skull surface, 0.5–0.7 mm anterior to bregma/0.3–0.5 mm from midline, 1.7–2.5 mm deep) and hippocampus (at 20° from vertical plane, 3.5–3.7 mm posterior to bregma/3.5–3.8 from midline, 1.2–1.7 mm deep). Both LFP and multi-unit activity (MUA) were recorded for at least 60 min at a sampling rate of 32 kHz using a multi-channel extracellular amplifier (Digital Lynx 4S with no gain, Neuralynx, Dublin, Ireland) and *Cheetah* acquisition software. Recorded signals were band-pass filtered (0.1 Hz to 5 kHz). For data analysis, channels were selected based on post-mortem histological investigation and the presence of specific activity patterns (PL: nested gamma spindle bursts (NGs) and high-frequency oscillations; hippocampus: reversal of LFP over stratum pyramidale was used to select channel with sharp-waves of minimum amplitude). Data were imported and analysed off-line using custom-written tools (*Matlab* software, Mathworks, Natick, MA). Detection and classification of discontinuous activity patterns were performed using a modified version of a previously described algorithm^75^ and confirmed by visual inspection.

### Behavioural experiments

*Dlgap4*^geo/geo^ and *Dlgap4*^*+*/+^ mice utilized for behavioural studies were offspring from heterozygous breeding pairs (mixed C57BL/6J × 129Ola genetic background, with >10 backcrosses into C57BL/6J). Three different mouse cohorts were used. To avoid a ‘litter effect’ within cohorts, no more than two animals per genotype came from the same litter. All tests were video-recorded. Cohort 2 consisted of male and female mice born and raised in a vivarium with a normal 12 h/12 h-light/dark cycle (lights on: 6:00 am) under standard housing conditions (21 ± 1°C, 40–50% humidity). Animals were used to assess vocal communication and reciprocal social interaction behaviour in juvenile mice. Experiments were executed during the dark cycle, in a room neighbouring the vivarium that was illuminated with dim red light. Tests started and ended at least 2 h after light offset/before light onset.

#### Interaction-induced USV and reciprocal social interaction behaviour in juvenile mice

To induce USV and measure reciprocal social interaction behaviour in juvenile mice (P26 ± 2), same-sex/same-genotype animal pairs consisting of non-littermates were allowed to socially interact for 5 min after one mouse had been habituated to the test environment for 1 min^47,76^. To enhance social motivation, mice were isolated for 24 h prior to testing. Testing was performed in a clean Makrolon Type III cage with fresh bedding. USV emission was monitored by an UltraSoundGate Condenser CM16 microphone (Avisoft Bioacoustics, Glienicke Germany) placed 15 cm above the cage floor. Mouse behaviour was video-taped throughout the test phase.

#### Acoustical recording and analysis

Acoustic data were recorded (sampling rate: 250,000 Hz, 16-bit format) using *Avisoft RECORDER USGH* software and an UltraSoundGate 116 USB audio device (Avisoft Bioacoustics, Glienicke Germany)^77,78^. For analysis, recordings were transferred to *Avisoft SASLab Pro* software (Avisoft Bioacoustics, Glienicke Germany) and a fast Fourier transform was conducted (512 FFT length, 100% frame, Hamming window and 75% time window overlap). Spectrograms were produced at 488 Hz of frequency and 0.512 ms of time resolution. Interaction-induced USVs were analysed interactively by an experienced observer to avoid false positives. Physical social interactions were scored by an experienced observer blinded to experimental conditions (*The Observer* software, Noldus Information Technology, Solingen, Germany)^47^. Total number of USV and physical social interactions was calculated in 1 min time bins.

For cohorts 1 and 3 *Dlgap4*^+/+^ mice and *Dlgap4*^geo/geo^ 10-week-old male littermates were transferred from the breeding facility into a vivarium with an inverted 12 h/12 h-light/dark cycle (lights off at 8:00 am) and maintained under standard housing conditions. Behavioural tests were executed with 12–20-week-old mice during the dark cycle in a room neighbouring the vivarium. Tests started and ended at least 2 h after light offset and 3 h before light onset, respectively. The experimental material was cleaned with soap, water and ethanol (70%) before and after each contact with an animal. Cohort 2 was used to assess direct reciprocal social interaction of adult mice.

#### Direct reciprocal social interaction

Male mice from cohort 3 underwent the reciprocal social interaction test 3 days after being single housed in a Macrolon type II cage (13 × 20 × 23 cm). The home cage was gently transferred from the vivarium to the experimental room. A Plexiglas panel provided with ventilation holes substituted the cage top. After 5 min, a C57Bl/6J age- and body-weight-matched unfamiliar male (intruder) was introduced in the cage of the focal animal (resident). The test lasted for 10 min after the first resident–intruder contact. Social interactions were analysed using *The Observer* software. Video-recorded behaviour was analysed by a trained experimenter blind to the genotype. The experimenter trained himself until repeatedly scoring at least 90% of consistency between two analyses of the same mouse performed at different times (calculated using *Reliability Test* of *The Observer* software; maximal time discrepancy between two evaluations: 1 s).

Mice in cohort 1 were used for behavioural tests performed in the following order: open field, EPM, spontaneous alternation, water maze, circadian activity. Video records were used to generate tracks representing the position of mice. Tracks were analysed with *EthoVision* software (Noldus Information Technology, Solingen, Germany)^79^.

#### Open field

The open field test was performed in a box (50 × 50 cm and 40 cm high) illuminated with white light (10 lux). Mice were placed in one corner of the box and their behaviour was analysed for 15 min. Distance moved, mean minimal distance to wall, time spent in the centre (an imaginary 25 × 25 cm square in the middle of the arena) were analysed with *EthoVision* software, whereas the parameters rearing on wall (mouse stands on hind limbs and touches wall with at least one forepaw) and self-grooming were analysed with *The Observer*.

#### Elevated plus maze

The maze has the shape of a plus with four 30 cm long and 5 cm wide arms, connected by a squared centre (5 × 5 cm). Two opposing arms are bordered by 15 cm high walls (closed arms), whereas the other two arms (open arms) are bordered by a 2 mm rim. The maze was elevated 75 cm from the floor and an infrared camera allowed video-recording. The mouse was placed into the centre facing one open arm and left on the maze for 5 min. The following parameters were analysed with *The Observer*: entries into open and closed arms (calculated when all four paws were on an arm), total transitions (sum of entries into open and closed arms), entries into edges of open arms (calculated when mouse reaches with its snout the edge of an open arm), latency to enter into open arms, latency to reach edge of an open arm, stretch attend posture towards open arm, rearing, self-grooming, head dips from ‘protected’ area (head movements over side of an open arm with the snout pointing downwards while the mouse remains in the centre or closed arm), head dips from ‘unprotected’ area (head dips are done as the mouse is on the open arms) and self-grooming.

#### Spontaneous alternation

The maze consists of three equally sized arms (34 × 5 × 30 cm) made of transparent Plexiglas connected such as to make a Y and illuminated with 5 lux. Mice were placed in the centre of the maze and allowed to freely explore the maze until performing 27 transitions or after a maximal time of 20 min. An entry into any arm with the four paws was considered as transition. An entry into a new arm after having visited the two other arms was considered as alternation. Data were analysed as a percentage of alternations over all transitions. Average time to make a transition was calculated by dividing the duration of the test by the number of total transitions.

#### Water maze

The water maze consists of a circular tank (145 cm in diameter) circled by dark curtains. The water was made opaque by the addition of non-toxic white paint such that the white platform (14 cm diameter, 9 cm high, 1 cm below water surface) was not visible. Four dark landmarks (35 × 35 cm) placed on a white background, differing in shape and grey gradient were hung on the inner white wall of the maze. Light was provided by four white spotlights placed on the floor around the pool to provide homogeneous illumination of 60 lux. Before the experiment started, mice were familiarized for 3 days to swim and climb onto a platform (diameter of 10 cm) placed in a small rectangular maze (42.5 × 26.5 cm and 15.5 cm high). During familiarization, the position of the platform was unpredictable since its location was randomized and training was performed under darkness. After familiarization, mice underwent 3 learning days during which they had to learn the location of a hidden platform. Starting position and position from which mice were taken out of the maze were randomized. At day 1, mice underwent four learning trials (maximum duration 90 s, inter-trial interval of 20 min). After staying on the platform for 10 s, mice were returned to their home cage and warmed up under red light. Day 2 consisted of five trials: trials 1, 2, 4 and 5 were learning trials, whereas trial 3 was a so-called probe trial during which the platform was removed (short-term memory probe trial). Day 3 consisted of one probe trial (long-term memory probe trial). The probe trials had a fixed duration of 60 s. All trials were video-recorded and the position of the mice was tracked using *EthoVision* software.

#### Circadian activity

Circadian activity of single-housed mice was measured for 1 week. It was monitored using the infrared sensor *Mouse-E-Motion* (Infra-e-motion, Hamburg, Germany)^79^. Mouse activity is shown as a percentage of time that the mouse was moving.

### Experimental design and statistical analysis

To decide the sample size for our behavioural, electrophysiological and biochemical experiments, we followed the standard sample sizes used in similar experiments in each of the relevant fields in the literature. Data generated in entirely independent experiments were analysed with a two-sided paired Student’s *t*-test (level of significance *α* = 0.05). Three different mouse cohorts were used for distinct sets of experiments. To avoid a ‘litter effect’ within each cohort, no more than two animals per genotype came from the same litter. Knockout and wildtype animals are offspring of heterozygous breeding pairs. All behavioural data were analysed either by *t*-test or by mixed two-way ANOVA for repeated measurements having genotype as between-group factor and time interval (for the open field test) or trial (for the water maze test) as within-group factor, followed by Newman–Keuls post-hoc analyses when appropriate. All tests were two-tailed and significance level was set at *p* < 0.05. For each experiment including statistical analysis, the statistical tests used, exact values of *N*, definitions of center, methods of multiple test correction, and dispersion and precision measures as well as exact *p*-values are provided in the corresponding figure legend. For every figure, the used statistical tests are justified as appropriate and the data meet the assumptions of the test. The results reported in this study are the aggregate of multiple independent experiments, demonstrating the reproducibility of the findings. In all experimental analyses performed for this study, we did not encounter any outliers. Thus, no data, samples or animals were excluded for statistical analysis. For all tests, including animal studies, no methods of randomization were used. Investigators analysing data were blinded for the analysis of dendritic arborisation, spine shape and density, synapse density, synaptic vesicle density and PSD size analysis, in vivo brain electrophysiological examination, behavioural tests and USV studies. All behavioural tests were video-recorded and sampled automatically if possible, e.g. total distance moved in open field study or body movements for the analysis of circadian activity. Researchers were not blinded for all other than the above-mentioned behavioural tests, Northern/Western blot analyses, pathological examinations and in vitro electrophysiology.

## References

[1] Chua JJ, Kindler S, Boyken J, Jahn R (2010). The architecture of an excitatory synapse. J. Cell Sci..

[2] Monteiro P, Feng G (2017). SHANK proteins: roles at the synapse and in autism spectrum disorder. Nat. Rev. Neurosci..

[3] Sheng M, Kim E (2011). The postsynaptic organization of synapses. Cold Spring Harb. Perspect. Biol..

[4] Verpelli C, Schmeisser MJ, Sala C, Böckers TM (2012). Scaffold proteins at the postsynaptic density. Adv. Exp. Med. Biol..

[5] Bourgeron T (2015). From the genetic architecture to synaptic plasticity in autism spectrum disorder. Nat. Rev. Neurosci..

[6] de la Torre-Ubieta L, Won H, Stein JL, Geschwind DH (2016). Advancing the understanding of autism disease mechanisms through genetics. Nat. Med..

[7] De Rubeis S (2014). Synaptic, transcriptional and chromatin genes disrupted in autism. Nature.

[8] Jiang YH, Ehlers MD (2013). Modeling autism by SHANK gene mutations in mice. Neuron.

[9] Pinto D (2010). Functional impact of global rare copy number variation in autism spectrum disorders. Nature.

[10] Torres VI, Vallejo D, Inestrosa NC (2017). Emerging synaptic molecules as candidates in the etiology of neurological disorders. Neural Plast..

[11] Leblond CS (2014). Meta-analysis of SHANK mutations in autism spectrum disorders: a gradient of severity in cognitive impairments. PLoS Genet..

[12] Chien WH (2013). Deep exon resequencing of DLGAP2 as a candidate gene of autism spectrum disorders. Mol. Autism.

[13] Chien WH (2010). Identification and molecular characterization of two novel chromosomal deletions associated with autism. Clin. Genet..

[14] Crane J (2011). Family-based genetic association study of DLGAP3 in Tourette Syndrome. Am. J. Med. Genet. B Neuropsychiatr. Genet..

[15] Li J (2015). An association study between DLGAP1rs11081062 and EFNA5 rs26728 polymorphisms with obsessive-compulsive disorder in a Chinese Han population. Neuropsychiatr. Dis. Treat..

[16] Li JM (2014). Role of the DLGAP2 gene encoding the SAP90/PSD-95-associated protein 2 in schizophrenia. PLoS One.

[17] Ryu S (2011). Interaction between genetic variants of DLGAP3 and SLC1A1 affecting the risk of atypical antipsychotics-induced obsessive-compulsive symptoms. Am. J. Med. Genet. B Neuropsychiatr. Genet..

[18] Sagar A (2017). De novo unbalanced translocation (4p duplication/8p deletion) in a patient with autism, OCD, and overgrowth syndrome. Am. J. Med. Genet. A.

[19] Zuchner S (2009). Multiple rare SAPAP3 missense variants in trichotillomania and OCD. Mol. Psychiatry.

[20] Kindler S, Rehbein M, Classen B, Richter D, Böckers TM (2004). Distinct spatiotemporal expression of SAPAP transcripts in the developing rat brain: a novel dendritically localized mRNA. Brain Res. Mol. Brain Res..

[21] Welch JM, Wang D, Feng G (2004). Differential mRNA expression and protein localization of the SAP90/PSD-95-associated proteins (SAPAPs) in the nervous system of the mouse. J. Comp. Neurol..

[22] Takeuchi M (1997). SAPAPs. A family of PSD-95/SAP90-associated proteins localized at postsynaptic density. J. Biol. Chem..

[23] Welch JM (2007). Cortico-striatal synaptic defects and OCD-like behaviours in Sapap3-mutant mice. Nature.

[24] Ade KK (2016). Increased metabotropic glutamate receptor 5 signaling underlies obsessive-compulsive disorder-like behavioral and striatal circuit abnormalities in mice. Biol. Psychiatry.

[25] Wan Y, Feng G, Calakos N (2011). Sapap3 deletion causes mGluR5-dependent silencing of AMPAR synapses. J. Neurosci..

[26] Jiang-Xie LF (2014). Autism-associated gene Dlgap2 mutant mice demonstrate exacerbated aggressive behaviors and orbitofrontal cortex deficits. Mol. Autism.

[27] Coba MP (2018). Dlgap1 knockout mice exhibit alterations of the postsynaptic density and selective reductions in sociability. Sci. Rep..

[28] Wan Y (2014). Circuit-selective striatal synaptic dysfunction in the Sapap3 knockout mouse model of obsessive-compulsive disorder. Biol. Psychiatry.

[29] Stryke D (2003). BayGenomics: a resource of insertional mutations in mouse embryonic stem cells. Nucleic Acids Res..

[30] Feng G (2000). Imaging neuronal subsets in transgenic mice expressing multiple spectral variants of GFP. Neuron.

[31] Golovyashkina N, Sündermann F, Brandt R, Bakota L (2014). Reconstruction and morphometric analysis of hippocampal neurons from mice expressing fluorescent proteins. Neuromethods.

[32] Penazzi L (2016). Abeta-mediated spine changes in the hippocampus are microtubule-dependent and can be reversed by a subnanomolar concentration of the microtubule-stabilizing agent epothilone D. Neuropharmacology.

[33] Sündermann, F., Golovyashkina, N., Tackenberg, C., Brandt, R. & Bakota, L. High-resolution imaging and evaluation of spines in organotypic hippocampal slice cultures, Vol. 846. in *Methods in Molecular Biology* (ed Skaper, S. D.) 277–293 (Humana Press, New York City, 2012). 10.1007/978-1-61779-536-7_24 22367819

[34] Paoletti P, Bellone C, Zhou Q (2013). NMDA receptor subunit diversity: impact on receptor properties, synaptic plasticity and disease. Nat. Rev. Neurosci..

[35] Brockmann MD, Poschel B, Cichon N, Hanganu-Opatz IL (2011). Coupled oscillations mediate directed interactions between prefrontal cortex and hippocampus of the neonatal rat. Neuron.

[36] Hartung H, Brockmann MD, Poschel B, De Feo V, Hanganu-Opatz IL (2016). Thalamic and entorhinal network activity differently modulates the functional development of prefrontal–hippocampal interactions. J. Neurosci..

[37] Thiel CM, Müller CP, Huston JP, Schwarting RK (1999). High versus low reactivity to a novel environment: behavioural, pharmacological and neurochemical assessments. Neuroscience.

[38] Steimer T (2011). Animal models of anxiety disorders in rats and mice: some conceptual issues. Dialog. Clin. Neurosci..

[39] Guilmatre A, Huguet G, Delorme R, Bourgeron T (2014). The emerging role of *SHANK* genes in neuropsychiatric disorders. Dev. Neurobiol..

[40] Iasevoli F, Tomasetti C, de Bartolomeis A (2013). Scaffolding proteins of the post-synaptic density contribute to synaptic plasticity by regulating receptor localization and distribution: relevance for neuropsychiatric diseases. Neurochem. Res..

[41] Panksepp JB (2007). Affiliative behavior, ultrasonic communication and social reward are influenced by genetic variation in adolescent mice. PLoS One.

[42] Shipton OA, Paulsen O (2014). GluN2A and GluN2B subunit-containing NMDA receptors in hippocampal plasticity. Philos. Trans. R. Soc. Lond. B Biol. Sci..

[43] Sobczyk A, Scheuss V, Svoboda K (2005). NMDA receptor subunit-dependent [Ca2+] signaling in individual hippocampal dendritic spines. J. Neurosci..

[44] Bayer KU, De Koninck P, Leonard AS, Hell JW, Schulman H (2001). Interaction with the NMDA receptor locks CaMKII in an active conformation. Nature.

[45] Halt AR (2012). CaMKII binding to GluN2B is critical during memory consolidation. EMBO J..

[46] Silverman JL, Yang M, Lord C, Crawley JN (2010). Behavioural phenotyping assays for mouse models of autism. Nat. Rev. Neurosci..

[47] Wöhr M (2015). Lack of parvalbumin in mice leads to behavioral deficits relevant to all human autism core symptoms and related neural morphofunctional abnormalities. Transl. Psychiatry.

[48] Wöhr M, Scattoni ML (2013). Behavioural methods used in rodent models of autism spectrum disorders: current standards and new developments. Behav. Brain Res..

[49] Ameis SH (2011). Impaired structural connectivity of socio-emotional circuits in autism spectrum disorders: a diffusion tensor imaging study. PLoS One.

[50] Gogolla N, Takesian AE, Feng G, Fagiolini M, Hensch TK (2014). Sensory integration in mouse insular cortex reflects GABA circuit maturation. Neuron.

[51] Rothwell PE (2014). Autism-associated neuroligin-3 mutations commonly impair striatal circuits to boost repetitive behaviors. Cell.

[52] Zikopoulos B, Barbas H (2013). Altered neural connectivity in excitatory and inhibitory cortical circuits in autism. Front. Hum. Neurosci..

[53] Pehrs C, Samson AC, Gross JJ (2015). The quartet theory: implications for autism spectrum disorder: comment on “The quartet theory of human emotions: an integrative and neurofunctional model” by S. Koelsch et al. Phys. Life Rev..

[54] Blundell J (2010). Neuroligin-1 deletion results in impaired spatial memory and increased repetitive behavior. J. Neurosci..

[55] Etherton M (2011). Autism-linked neuroligin-3 R451C mutation differentially alters hippocampal and cortical synaptic function. Proc. Natl. Acad. Sci. U.S.A..

[56] Jamain S (2008). Reduced social interaction and ultrasonic communication in a mouse model of monogenic heritable autism. Proc. Natl. Acad. Sci. U.S.A..

[57] Peca J (2011). Shank3 mutant mice display autistic-like behaviours and striatal dysfunction. Nature.

[58] Radyushkin K (2009). Neuroligin-3-deficient mice: model of a monogenic heritable form of autism with an olfactory deficit. Genes Brain Behav..

[59] Schmeisser MJ (2012). Autistic-like behaviours and hyperactivity in mice lacking ProSAP1/Shank2. Nature.

[60] Sungur AO, Schwarting RK, Wöhr M (2016). Early communication deficits in the Shank1 knockout mouse model for autism spectrum disorder: developmental aspects and effects of social context. Autism Res..

[61] Tabuchi K (2007). A neuroligin-3 mutation implicated in autism increases inhibitory synaptic transmission in mice. Science.

[62] Wöhr M, Roullet FI, Hung AY, Sheng M, Crawley JN (2011). Communication impairments in mice lacking Shank1: reduced levels of ultrasonic vocalizations and scent marking behavior. PLoS One.

[63] Won H (2012). Autistic-like social behaviour in Shank2-mutant mice improved by restoring NMDA receptor function. Nature.

[64] Chen J, Yu S, Fu Y, Li X (2014). Synaptic proteins and receptors defects in autism spectrum disorders. Front. Cell. Neurosci..

[65] Mackowiak M, Mordalska P, Wedzony K (2014). Neuroligins, synapse balance and neuropsychiatric disorders. Pharmacol. Rep..

[66] Minocherhomji S (2014). Epigenetic remodelling and dysregulation of DLGAP4 is linked with early-onset cerebellar ataxia. Hum. Mol. Genet..

[67] Fuchs H (2011). Mouse phenotyping. Methods.

[68] Gailus-Durner, V. et al. Systemic first-line phenotyping, Vol. 530. in *Gene Knockout Protocols. Methods in Molecular Biology* (eds Wurst, W. & Kühn, R.) 463–509 (Humana Press, New York City, 2009). 10.1007/978-1-59745-471-1_25 19266331

[69] Koh IY, Lindquist WB, Zito K, Nimchinsky EA, Svoboda K (2002). An image analysis algorithm for dendritic spines. Neural Comput..

[70] Sawallisch C (2009). The insulin receptor substrate of 53 kDa (IRSp53) limits hippocampal synaptic plasticity. J. Biol. Chem.

[71] Coba MP (2009). Neurotransmitters drive combinatorial multistate postsynaptic density networks. Sci. Signal..

[72] Schütt J, Falley K, Richter D, Kreienkamp HJ, Kindler S (2009). Fragile X mental retardation protein regulates the levels of scaffold proteins and glutamate receptors in postsynaptic densities. J. Biol. Chem..

[73] Sambrook J, Fritsch EF, Maniatis T (1989). Molecular Cloning a Laboratory Manual.

[74] Plath N (2006). Arc/Arg3.1 is essential for the consolidation of synaptic plasticity and memories. Neuron.

[75] Cichon NB, Denker M, Grun S, Hanganu-Opatz IL (2014). Unsupervised classification of neocortical activity patterns in neonatal and pre-juvenile rodents. Front. Neural Circuits.

[76] Beis D (2015). Brain serotonin deficiency leads to social communication deficits in mice. Biol. Lett..

[77] Wöhr M, van Gaalen MM, Schwarting RK (2015). Affective communication in rodents: serotonin and its modulating role in ultrasonic vocalizations. Behav. Pharmacol..

[78] Mosienko V, Beis D, Alenina N, Wöhr M (2015). Reduced isolation-induced pup ultrasonic communication in mouse pups lacking brain serotonin. Mol. Autism.

[79] Freitag S, Schachner M, Morellini F (2003). Behavioral alterations in mice deficient for the extracellular matrix glycoprotein tenascin-R. Behav. Brain Res..

